# Prior metabolic surgery reduced COVID-19 severity: Systematic analysis from year one of the COVID-19 pandemic

**DOI:** 10.1016/j.heliyon.2023.e15824

**Published:** 2023-04-26

**Authors:** G. Craig Wood, Peter N. Benotti, Rodrigo M. Fano, James T. Dove, David DK. Rolston, Anthony T. Petrick, Christopher D. Still

**Affiliations:** a Obesity Institute, Geisinger, Danville, PA, USA; b Geisinger Commonwealth School of Medicine, Geisinger, Scranton, PA, USA; c Department of Medicine, Geisinger, Danville, PA, USA; d Department of Surgery, Geisinger, Danville, PA, USA

**Keywords:** Metabolic surgery, COVID-19, Outcomes, Pooled analysis

## Abstract

**Background:**

Obesity is a risk factor for COVID-19 severity. Recent studies suggest that prior metabolic surgery (MS) modifies the risk of COVID-19 severity.

**Methods:**

COVID-19 outcomes were compared between patients with MS (n = 287) and a matched cohort of unoperated patients (n = 861). Multiple logistic regression was used to identify predictors of hospitalization. A systematic literature review and pooled analysis was conducted to provide overall evidence of the influence of prior metabolic surgery on COVID-19 outcomes.

**Results:**

COVID-19 patients with MS had less hospitalization (9.8% versus 14.3%, p = 0.049). Age 70+, higher BMI, and low weight regain after MS were associated with more hospitalization after COVID-19. A systematic review of 7 studies confirmed that MS reduced the risk of post-COVID-19 hospitalization (OR = 0.71, 95%CI = [0.61–0.83], p < 0.0001) and death (OR = 0.44, 95%CI = [0.30–0.65], p < 0.0001).

**Conclusion:**

MS favorably modifies the risks of severe COVID-19 infection. Older age and higher BMI are major risk factors for severity of COVID-19 infection.

## Highlights

• Patients with metabolic surgery had improved COVID-19 outcomes.
• Pooled analysis of existing studies confirmed the improvement in COVID-19 outcomes.
• Older age and higher BMI were associated with increased COVID-19 hospitalization.
• Weight regain after metabolic surgery was associated with COVID-19 hospitalization.

## 1 Introduction

Since 2020, investigation into the worldwide spread of severe acute respiratory syndrome coronavirus 2 (SARS-Cov-2) has led to the general acceptance that obesity, visceral obesity as well as aspects of impaired metabolic heath are associated with increased risks for adverse outcomes complicating infection with SARS-CoV-2 [[1], [2], [3]]. More recently, larger population studies have confirmed these findings and demonstrated that obesity itself, even in the absence of comorbid diseases like diabetes predisposes to increased risk for adverse outcomes most notable in younger patients [4,5]. These discoveries have led to the addition of increased susceptibility to SARS-CoV-2 infection to the list of recognized complications of obesity [6].

The established association between obesity and poor clinical outcome of COVID-19 infection has contributed to the hypothesis that obesity might be a modifiable risk factor for severity of COVID-19 infection. The proven efficacy of metabolic and bariatric surgery in weight management and restoration of metabolic health have resulted in numerous large studies attempting to compare outcomes in patients with severe obesity who underwent metabolic surgery and contracted infection with COVID-19 during surgical weight loss [[7], [8], [9], [10], [11], [12], [13], [14], [15], [16], [17], [18], [19]]. The findings to date suggest that surgical weight loss is associated with improved outcomes such as fewer hospital admissions [7,10,13], less requirement for mechanical ventilation [9,12,13], and reduced mortality [10,12,15] in comparison to non-operated controls with severe obesity.

The purpose of this paper is to introduce additional evidence supporting improved COVID-19 outcomes for those with a history of metabolic surgery as well as to conduct a systematic review and meta-analysis of the studies to date which have addressed risk of hospitalization and death from COVID-19 infection in patients with severe obesity who underwent metabolic surgery compared with the severity in unoperated comparison group with severe obesity. In addition, this paper will investigate clinical factors in metabolic surgery patients which may contribute to the risk of hospitalization with COVID-19 infection.

## 2 Methods

### 2.1 Geisinger cohort

An established cohort of patients with metabolic surgery between 2003 and 2020 was followed throughout the first year of the COVID-19 pandemic (through April 2021). Prospectively obtained patient data within this metabolic surgery registry are regularly updated and maintained using their data from the electronic medical record and other data collected specifically for an Institutional Research Review Board approved study [20]. A COVID-19 database from the same institution was used to identify patients with a positive COVID-19 test (RT-PCR), whether they were hospitalized, and their hospitalization outcomes. The cohort with previous metabolic surgery followed by a positive COVID-19 test was selected for this study. Study data including baseline clinical characteristics, metabolic surgery outcomes including weight loss, and COVID-19 outcomes were extracted from the metabolic surgery registry. Additionally, chart review was used to validate when hospitalizations were COVID-19 related, to validate other COVID-19 outcomes, and to obtain additional data from chest imaging studies.

Electronic medical record data is used to maintain a separate registry of primary care patients that are eligible for metabolic surgery but haven’t had metabolic surgery. This cohort has been successfully used to identify a comparison group that was matched to the metabolic surgery patients and longitudinally studied for various health outcomes [[21], [22], [23]]. For this study, the COVID-19 positive patients with a history of metabolic surgery were matched to eligible COVID-19 positive patients that didn’t have metabolic surgery (in a ratio of 3 comparators: 1 case) without replacement using a propensity score that included age at time of metabolic surgery, sex, BMI at metabolic surgery, and selected risk factor status at time of metabolic surgery (smoking, diabetes, hypertension, hyperlipidemia, COPD, asthma, history of cancer, history of CHF, and chronic kidney disease). The comparison group was selected by first stratifying prospective candidates based on year of surgery (using 2004–2008, 2009–2012, 2013–2016, 2017–2018, 2019–2020; which roughly represent quintiles by year of surgery) then characterizing each potential comparison patient that was active in primary care during the stratified period and extracting the clinical risk factor status at that stratified time period (e.g. BMI for 2004–2008 was the median BMI during the years 2004–2008).

For the metabolic surgery group, COVID-19 outcomes including percent with any hospitalization (related or unrelated to COVID-19), hospitalization specifically related to COVID-19, use of chest imaging, any ICU stay, any ventilator use, and death were recorded. In this cohort, multiple logistic regression was used to identify predictors of any hospitalization and any COVID-19 related hospitalization. Factors considered for inclusion within the regression model included sex, age, race/ethnicity, BMI at time of metabolic surgery, BMI at time of COVID-19, maximum weight loss from metabolic surgery to COVID-19, weight regained from metabolic surgery (≥20% weight regained from nadir after bariatric surgery to COVID-19) [24], and diabetes status (never had diabetes, diabetes remission with metabolic surgery, and persistent diabetes from metabolic surgery to COVID-19). Items were selected for inclusion in a forward stepwise approach starting with the strongest predictor and adding additional items until no addition items were significant when added to the model.

Means with standard deviations and percentages were used to describe and contrast the metabolic surgery cohort and the matched comparison cohort. Chi-square test, Fisher’s exact test, and two sample t-tests were used to compare COVID-19 outcomes between the two cohorts. SAS version 9.4 was used for statistical analysis and p-values <0.05 were considered significant.

### 2.2 Pooled analysis

The aim of this systematic review and pooled analysis was to estimate the association of history of metabolic surgery and reduction in COVID-19 hospitalizations and death. Eligible studies (including the results included herein) were limited to those including both metabolic surgery cases and matched group of non-metabolic surgery patients that were positive for COVID-19 infection. Since hospitalization was the primary outcome, studies that were limited to hospitalized patients were excluded. An electronic search was performed on March 29, 2022, using PubMed.gov of the National Library of Medicine to derive a comprehensive list of studies potentially eligible for analysis. Using the “Advanced Search” function, the following search terms were utilized: (“COVID” OR “sarscov2” OR “sarscov-2” OR “coronavirus” OR “COVID-19”) AND (“bariatric” OR “RYGB” OR “gastric bypass” OR “sleeve” OR “gastrectomy”). The resulting abstracts and titles of the identified studies were screened for duplicates and relevance. Further review of relevant studies was conducted by reading the full text of potential studies to narrow down the list for final review. The reference lists within the subset of selected studies were scrutinized for additional studies that were not discovered in the electronic search. Data were extracted from each study including the overall number of patients within each group, the number that had a hospitalization, and the number with death. The Cochran-Mantel-Haenszel method was used to calculate pooled odds ratios and 95% confidence intervals for hospitalization and death. Homogeneity among studies was examined using Breslow-Day test. In cases where there were zero events (e.g. no COVID-19 deaths), a Haldane-Anscombe correction was implemented.

## 3 Results

### 3.1 Geisinger cohort

Of the 7109 with completed metabolic surgery between 1/1/2003 and 3/14/2020 (prior to start of COVID-19 restrictions), there were 5397 that had recent follow-up and 287 had a COVID-19 positive test between March 2020 and April 2021. These 287 included 232 with Roux-en-Y gastric bypass (RYGB), 37 with laparoscopic sleeve gastrectomy (LSG), 13 with biliopancreatic diversion with duodenal switch (BPD-DS), and 5 with adjustable gastric banding (Band). The 287 COVID-19 positive metabolic surgery cases and the 861 COVID-19 positive matched patients were similar regarding age, sex, BMI, and comorbidity status (Table 1).

**Table 1:** Descriptive analysis of Geisinger bariatric cases versus matched comparison group.

	History of metabolic surgery (N = 287)	Matched comparison group (n = 861)
Mean age (SD)	43.7 (11.0)	44.4 (13.4)
% female	85.0% (n = 244)	87.7% (n = 755)
Mean BMI (SD)	46.4 (7.9)	45.3 (6.8)
% diabetes	22.3% (n = 64)	21.7% (n = 187)
% hypertension	43.6% (n = 125)	44.1% (n = 380)
% hyperlipidemia	32.1% (n = 92)	32.5% (n = 280)
% COPD	3.5% (n = 10)	3.3% (n = 28)
% asthma	15.7% (n = 45)	16.4% (n = 141)
% cancer history	2.1% (n = 6)	2.0% (n = 17)
% CHF	1.7% (n = 5)	0.9% (n = 8)
% CKD	1.1% (n = 3)	0.9% (n = 8)
% smoker	39.0% (n = 112)	37.8% (n = 325)

As compared to those with a history of metabolic surgery, the non-operated matched comparison group had a higher percent with any hospitalization (14.3% versus 9.8%, p = 0.049), a higher percent with COVID-19 related hospitalization (12.3% versus 7.3%, p = 0.020), and a higher percent with chest imaging performed (13.0% versus 7.7%, p = 0.015). The percent with ICU stay and death was higher in the comparison group, but the differences were not significant (Table 2). The results of the chest imaging studies were examined for several outcomes (e.g. ground glass opacity, interlobar septal thickening, patchy airspace, etc.) but these results were not significantly different between groups.

**Table 2:** COVID-19 outcomes in history of bariatric surgery group versus matched comparison group.

	History of metabolic surgery (N = 287)	Matched comparison group (n = 861)	p-value
Any hospitalization	9.8% (n = 28)	14.3% (n = 123)	0.049
COVID hospitalization∗	7.3% (n = 21)	12.3% (n = 106)	0.020
Any chest imaging study (CT or Xray)	7.7% (n = 22)	13.0% (n = 112)	0.015
Any ICU stay	2.4% (n = 7)	4.3% (n = 37)	0.156
Any ventilator use	2.1% (n = 6)	2.1% (n = 18)	0.999
Death	1.7% (n = 5)	2.3% (n = 20)	0.559

∗ Per chart review.

Among the COVID-19 positive patients in the metabolic surgery cohort, there were several factors identified that were associated with any hospitalization and COVID-19 related hospitalization after COVID-19 infection (Table 3). Those age 70+ were about 11 times more likely to have any hospitalization (OR = 11.78, 95% CI = [2.73, 50.78], p = 0.0009) and about 18 times more likely to have a COVID-19 related hospitalization (OR = 18.74, 95% CI = [4.25, 82.61], p = 0.0001). Compared to BMI<30 kg/m^2^ at time of COVID-19, higher levels of BMI were associated with greater chance of any hospitalization (BMI 30–39: OR = 5.18, 95% CI = [1.04, 25.82], p = 0.045; BMI 40+: OR = 11.92, 95% CI = [1.92, 74.16], p = 0.0079). A similar, smaller trend was observed for COVID-19 related hospitalization, but this was not significant. Those without weight regain were more likely to have any hospitalization (OR = 5.17, p = 0.0050) but this was weaker and not significant for COVID-19 related hospitalization. Although those with persistent diabetes from metabolic surgery to COVID-19 had almost a 3 times greater chance of hospitalization, this was not significant in the multiple regression models.

**Table 3:** Multiple logistic regression results for any hospitalization and COVID-19 related hospitalization within the group that had a history of metabolic surgery (n = 287).

Parameter	Any hospitalization			COVID-19 related hospitalization		
	OR	95% CI	p-value	OR	95% CI	p-value
**Age at COVID-19**						
<60	Reference			Reference		
60–69	2.82	[0.89, 8.96]	0.079	3.46	[0.95, 12.61]	0.060
70+	11.78	[2.73, 50.78]	0.0009	18.74	[4.25, 82.61]	0.0001
**BMI at COVID-19**						
<30	Reference			Reference		
30–39	5.18	[1.04, 25.82]	0.045	3.20	[0.59, 17.33]	0.178
40+	11.92	[1.92, 74.16]	0.0079	5.93	[0.89, 39.37]	0.065
Weight regain*						
Yes (≥20%)	Reference			Reference		
No (<20%)	5.17	[1.64, 16.26]	0.0050	2.76	[0.78, 9.73]	0.115
**Diabetes status**						
No diabetes at COVID	Reference			Reference		
Persistent diabetes	2.84	[0.91, 8.91]	0.073	2.95	[0.85, 10.18]	0.088

∗ From nadir after bariatric surgery to COVID-19.

### 3.2 Pooled analysis

**Fig. 1:**
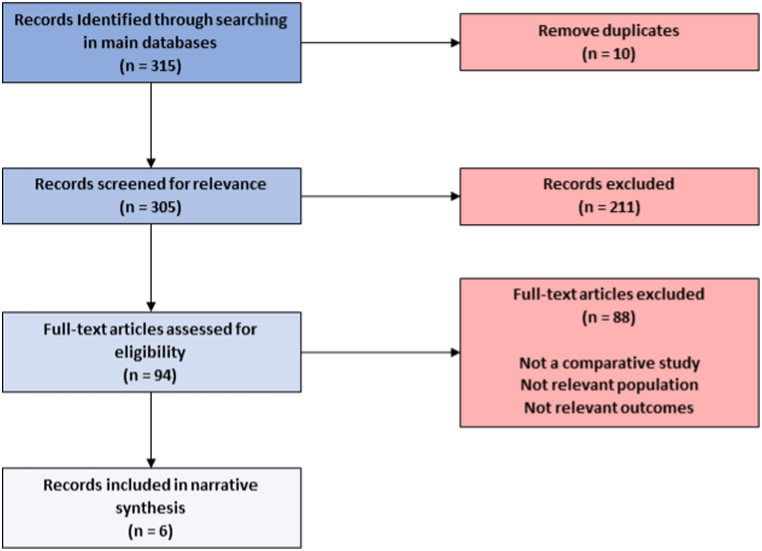
Flowchart for electronic literature search of studies evaluating relationships between prior metabolic surgery and COVID-19 outcomes.

**Fig. 2:**
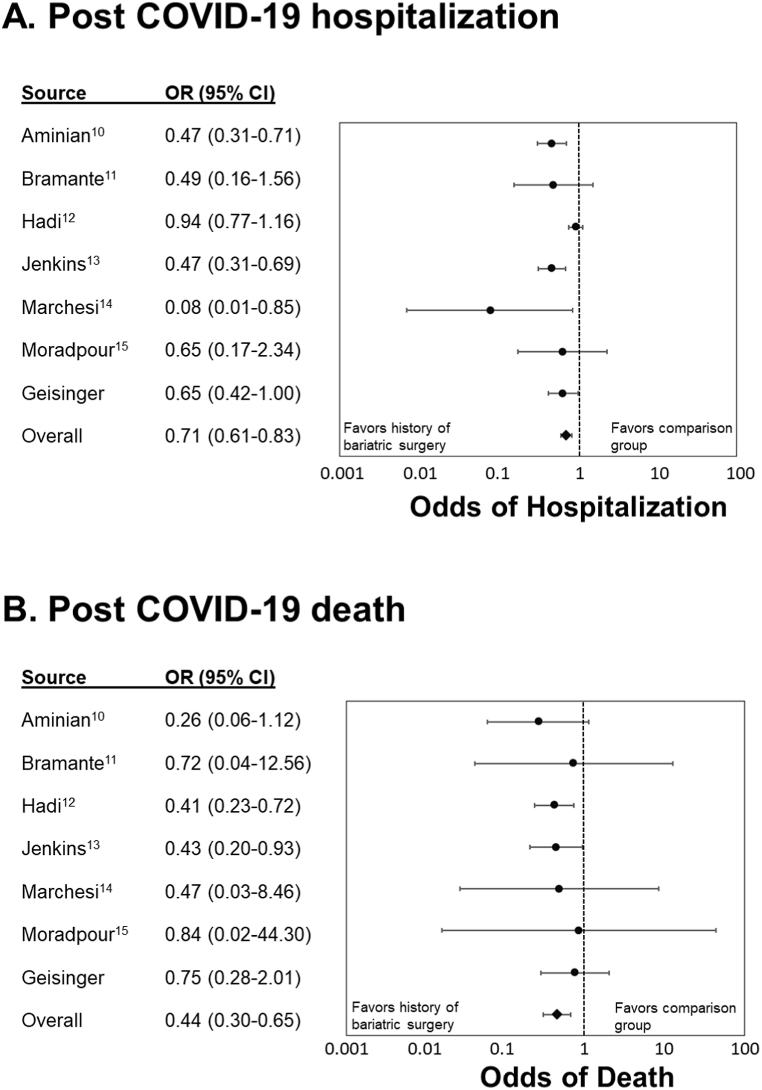
Forest plot for pooled analysis comparing post COVID-19 hospitalization (panel A) and death (panel B) between those with prior metabolic surgery and a matched non-operated comparison group.

The literature search yielded a total of 315 publication articles, of which 10 were excluded due to duplication. The remaining 305 articles were screened for relevance, resulting in the discarding of 211 articles. The remaining 94 articles were further examined of which 88 were excluded because they were not a comparative study, they didn’t include the same population, and/or they didn’t include the relevant outcomes. The remaining 6 were retained for the pooled analysis (Fig. 1) [[10], [11], [12], [13], [14], [15]]. These 6 studies were combined with the study results described above for a total of 7 studies including a total of 2618 with a history of metabolic surgery and 4272 non-metabolic surgery comparators. All studies reported both the hospitalization and death outcomes. The risk of hospitalization was reduced in the metabolic surgery group (OR = 0.71, 95% CI = [0.61, 0.83], p < 0.0001, Fig. 2). However, heterogeneity was detected when pooling results across studies (Breslow-Day p-value = 0.0026). In sensitivity analysis, each study was removed one at a time to evaluate homogeneity of the pooled odds ratio. When the data from Hadi were removed,^12^ the Breslow-Day test was not significant (p = 0.487) and the pooled odds ratio for hospitalization was 0.510 (95% CI = [0.40, 0.64], p < 0.0001). The test for lack of homogeneity of the pooled odds ratio for death was not significant (Breslow-Day test p = 0.928) and the risk of death was reduced in the metabolic surgery group (OR = 0.44, 95% CI = [0.30, 0.65], p < 0.0001, Fig. 2).

## 4 Discussion

These findings confirm that the risk of severe COVID-19 infection is reduced by metabolic surgery related weight loss. Severe obesity is a modifiable disease which is an established risk factor for severe COVID-19. Weight loss associated with metabolic surgery reduces this risk. When combining the results of this study’s 287 COVID patients and the 861 tightly matched non-operated controls with COVID-19 with 6 independent studies (including another 2331 metabolic surgery patients and another 3411 non-operative controls), COVID-19 hospitalization was reduced by 30% and mortality after COVID-19 infection was cut in half.

Unique findings in this study include the investigation of patient-related factors which are associated with COVID-19 outcomes in postoperative metabolic surgery patients. Age 70 and over in these patients was very strongly associated with any hospitalization and with COVID-related hospitalization. Also, a higher BMI was associated with an increased hospitalization risk as was a weight loss trajectory without the usual later weight regain, which raises the question of specific nutritional issues. Diabetes is a known risk factor for infection with COVID-19, and persistent diabetes after metabolic surgery was associated with a non-statistically significant 3-fold increase in the rate of hospitalizations. Additional study is needed to determine the impact of BMI, extent of weight loss with subsequent weight regain, and persistent diabetes on COVID-19 infection severity risk after metabolic surgery.

The established association in patients with severe obesity, particularly visceral obesity with the development of hypertension, cardiovascular disease, type 2 diabetes, and many cancers is a known contributor to COVID-19 infection severity [25]. In addition, multiple large population studies with outcomes adjusted for the comorbid disease burden together with meta-analyses have provided strong evidence that obesity is an independent risk factor for the severity of infection with COVID-19 [1,3,5]. The adult respiratory distress syndrome, a major cause of mortality with COVID*-*19 infection, is more common in patients with obesity [26]. Previous investigations have shown that individuals with obesity are at increased risk for morbidity and mortality with infection with influenza [27]. The findings that obesity is linked to increased severity of COVID-19 infection adds to the obesity disease burden [6].

Although the precise mechanisms which underly the increasing severity of COVID-19 infection in patients with obesity are unknown, evidence-based proposed mechanisms are emerging. The presence of a state of chronic immune activation and low-grade inflammation presumably originating in expanded adipose tissue resulting in increased levels of inflammatory and decreased levels of anti-inflammatory mediators, insulin resistance, and hyperleptinemia leads to abnormalities of both the innate and adaptive immune systems and endothelial dysfunction [28,29]. COVID-19 invades cells by binding to the angiotensin-converting enzyme-2 (ACE-2) receptor which is heavily expressed in adipose tissue and pulmonary alveolar cells [30,31]. The association between obesity and abnormalities of pulmonary function which include pulmonary restriction, reduced lung volumes, and ventilation/perfusion mismatch which predisposes to hypoxia as well as with obstructive sleep apnea is well known. Autopsy studies in patients dying of hypoxemic respiratory failure demonstrate diffuse alveolar damage and endothelial damage with micro thrombosis [32], and the thrombotic complications associated with COVID-19 are well described [33]. It is likely that the known association between obesity, thrombosis and endothelial dysfunction contribute to the development of these lesions [34].

The favorable impact of metabolic surgery on the risk of COVID-19 severity shown here is supported by the established physiological benefits of metabolic surgery which include a resolution of chronic low-grade systemic inflammation [35], improvement in pulmonary function [36], reduction in the pro-thrombotic potential [37], and improved endothelial function [38]. In addition, recent evidence suggests that patients who underwent metabolic surgery had fewer unplanned hospital admissions during short-midterm follow-up for infection when compared to non-operated controls [39].

## 5 Strengths and limitations

An important limitation of this trial is the susceptibility to confounding factors which can complicate retrospective matched cohort analyses. In addition, the data analysis is limited to the first year of the COVID pandemic when vaccines were not widely available. The impact of vaccines on hospitalizations and death can’t be determined in the present study. Although a sub-analysis of COVID-19 related hospitalizations was conducted, the severity of COVID-19 symptoms weren’t available to provide a more thorough analysis of patient management related to COVID-19.

The strengths of this study relate to the awareness that meta-analysis provides a high level of evidence influencing clinical practice. The clinical metabolic surgery registry utilized for this single institution study is a validated and vetted database which has been the data source for many clinical studies. Prior research matched metabolic surgery cases to a non-operated comparison group based on patient characteristics at the time of COVID-19 infection, which may fail to capture the full complement of the effect of metabolic surgery. However, this study improved upon the prior research by matching based on patient characteristics at the time of metabolic surgery. Finally, this is the first study that we are aware of which attempts to define clinical predictors of severe COVID-19 infection among patients who previously underwent metabolic surgery.

These findings support the accumulating evidence that obesity is a modifiable disease, and that COVID-19 should be added to the comorbid conditions improved by metabolic surgery [40].

## 6 Conclusions

Prior metabolic surgery reduced the risk of COVID-19 hospitalizations and COVID-19 deaths.

## Author contribution statement

Craig Wood and Peter Benotti: Conceived and designed the experiments; Analyzed and interpreted the data; Wrote the paper.

Rodrigo Fano: Performed the experiments, Wrote the paper.

James Dove: Analyzed and interpreted the data; Wrote the paper.

David Rolston, Anthony Petrick and Chris Still: Conceived and designed the experiments; Wrote the paper.

## Data availability statement

Data will be made available on request.

## Financial disclosures and source of funding

None of the authors have a financial interest in any of the products, devices, or drugs mentioned in this manuscript. Funding was obtained through internal institutional funds.

## Additional information

No additional information is available for this paper.

## Declaration of competing interest

There are no conflicts of interest for any authors.
